# Exome Capture with Heterologous Enrichment in Pig (*Sus scrofa*)

**DOI:** 10.1371/journal.pone.0139328

**Published:** 2015-10-02

**Authors:** Denis Guiatti, Elena Pomari, Slobodanka Radovic, Alessandro Spadotto, Bruno Stefanon

**Affiliations:** 1 Dipartimento di Scienze Agrarie ed Ambientali, Università degli Studi di Udine, Udine, Italy; 2 IGA Technology Services Srl, Parco Scientifico e Tecnologico L. Danieli, Udine, Italy; University of Bologna, ITALY

## Abstract

The discovery of new protein-coding DNA variants related to carcass traits is very important for the Italian pig industry, which requires heavy pigs with higher thickness of subcutaneous fat for Protected Designation of Origin (PDO) productions. Exome capture techniques offer the opportunity to focus on the regions of DNA potentially related to the gene and protein expression. In this research a human commercial target enrichment kit was used to evaluate its performances for pig exome capture and for the identification of DNA variants suitable for comparative analysis. Two pools of 30 pigs each, crosses of Italian Duroc X Large White (DU) and Commercial hybrid X Large White (HY), were used and NGS libraries were prepared with the SureSelectXT Target Enrichment System for Illumina Paired-End Sequencing Library (Agilent). A total of 140.2 M and 162.5 M of raw reads were generated for DU and HY, respectively. Average coverage of all the exonic regions for *Sus scrofa* (ENSEMBL Sus_scrofa.Sscrofa10.2.73.gtf) was 89.33X for DU and 97.56X for HY; and 35% of aligned bases uniquely mapped to off-target regions. Comparison of sequencing data with the Sscrofa10.2 reference genome, after applying hard filtering criteria, revealed a total of 232,530 single nucleotide variants (SNVs) of which 20.6% mapped in exonic regions and 49.5% within intronic regions. The comparison of allele frequencies of 213 randomly selected SNVs from exome sequencing and the same SNVs analyzed with a Sequenom MassARRAY® system confirms that this “human-on-pig” approach offers new potentiality for the identification of DNA variants in protein-coding genes.

## Introduction

For PDO (Protected Designation of Origin) ham production, the Italian pig industry requires heavy pigs, reared for 9 months and slaughtered at 160 kg live weight and with a carcass lean percentage ranging from 40% to 55%. According to the production guidelines (EC No: IT-PDO-0117-01149) [1], pigs must belong to the Large White and Landrace breeds, as approved by the Italian Genealogical Register, and Duroc crossbreeds. Italian pig industry for PDO hams requires heavy animals selected to have a higher amount of fat in comparison to those reared for fresh meat consumption, which are leaner and slaughtered at a lower age and live weight [2].

The genetic diversity of pig breeds and the associations with productive and functional traits have recently been published with the aim of describing the variability of genome for management and selection purposes. [3]. The recent availability of high density SNP (single nucleotide polymorphism) arrays [4] provides a powerful tool for association studies and provides standardized data which can be used for comparisons among researches, in addition to now being relatively inexpensive and easy to use. However, the application of these platforms neither offers the opportunity to expand the association studies to DNA variants not included in the array, nor to discover novel variants.

The next generation sequencing (NGS) techniques can provide a very large amount of genomic information at a reduced cost, enabling the sequencing of several animal genomes and the identification of new mutations. However, for association studies of quantitative traits a large number of individuals are required and, in the case of whole genome sequencing, costs and computational resources can be a limit to this approach. Among the NGS tools available to unravel genomic information, exome capture offers the opportunity to focus on regions of DNA that are potentially related to the expression of genes and proteins [5].

The discovery of new protein-coding DNA variants related to carcass traits is very important for pig production. Since Italian breeding schemes are targeted to select boars and sows with favorable characteristics for PDO ham production, like San-Daniele ham, exome capture can provide information for the targets of the Italian pig-breeding industry as carcass and thigh weight, back fat thickness and lean percentage. First exome capture sequencing for domestic *Sus scrofa* has been recently published [6], with the aim to offer new potentialities for the identification of DNA variants in protein coding genes which can be used for the study of biodiversity and for the selection of phenotypic traits of relevance. Since this published method was not commercially available when the present research begun, a heterologous enrichment for exome capture was used, a system already applied for SNP discovery in taxonomically closely related species [7]. In this methodological approach, the most limiting step is probably the enrichment phase, since its efficiency depends on the homology between probes and the target genome. In this study, we used a human commercial target enrichment kit to perform the capture and resequencing of the pig’s exome regions and to evaluate its performances for the identification of novel DNA variants.

## Materials and Methods

### Animals

For the research, we used 30 pigs, crosses of Italian Duroc X Large White (DU), and 30 pigs, crosses of Commercial hybrid X Large White (HY). The Duroc boars and the Large White sows belonged to genetically pure lines selected by National Association of Pig Breeders (ANAS, Rome, Italy).

The DU and HY crosses were reared on a commercial farm from birth to a final live weight greater than 154 kg, providing that the pigs were older than 270 days, following the requirements of the “Prosciutto di San Daniele” PDO. Individual live weights of pigs at weaning (average 29 days), at 10 weeks and at the end of the productive cycle were recorded and the average daily weight gain was calculated (S1 Table).

Animals were slaughtered in a local abattoir according to national legislation and carcass weight, lean percentage, back fat and loin thickness were recorded with a Fat-O-Meater instrument. Overall, a total number of 1032 DU and 1011 HY individual data were collected and used to compare the productive characteristics of the two crosses (S2 Table). For each cross of previously analyzed animals, sets of 30 individuals constituted of 5 pigs from 6 different boars, were selected.

### Ethics Statement

No animal was killed for the purpose of this study and no intervention on live pigs was carried out as a part of this study. Data collected on live animals during the growing phase were part of the periodical controls in the farms and no unrequired manipulation of animals which could cause pain, stress and any form of psychological and physical suffering was applied. No interaction with live animals was present and tissue sampling was performed on the carcasses. All data were collected from the farms and from the abattoir records.

Farmers and farms’ veterinary practitioners gave an informed consent to the animal study.

Animals were housed on commercial farms, which adhered to a high standard of veterinary care based on the best-practice manual and were under the control of the Italian Official Veterinary Service (ASL, N° 6, “Friuli Occidentale”) for the accomplishment of European and Italian animal welfare laws and directives. Animals had free access to clean water and were fed regularly to satisfy nutrient requirements. Space availability and bedding allowed the animals to express the species-specific behavioral repertoire. Animals were slaughtered in a commercial abattoir (Salumificio F.lli Uanetto & C. S.n.c., Ss Napoleonica—33050 Castions di Strada, Udine, Italy) under the supervision of a Veterinary officer of the Italian Official Veterinary Service, according to the European and Italian animal welfare laws and legislation. Samples and data collection was carried out between 2010 and 2012. No authorization by the ethical committee was requested, because it is not provided for by Italian law in such cases.

### DNA extraction

Muscle samples were collected at the abattoir and immediately frozen at -20°C. Extraction of DNA from the sample was performed using standard methods. DNA was isolated from muscle material (~40 mg), ground and washed twice by centrifugation (10000 g, 5 min) in 1 ml buffer (10 mM Tris HCl, 1 mM EDTA, pH 8.0). After removing the supernatant, the residue was suspended in 700 μl lysis buffer (50 mM Tris HCl, 200 mM NaCl, 20 mM EDTA, 1% sodium dodecyl sulfate (SDS), pH 8.0) with the addition of 25 μl proteinase K (10 mg/ml) and incubated on a water bath for 9–12 h at 47°C. Extraction of DNA was carried out by the phenol—chloroform method with the addition of a stage of treatment with ribonuclease A (0.1 mg/specimen, 1.5 h, 47°C) [8]. Sigma-Aldrich reagents were used at all DNA extraction stages. Complying with DNA quality requirements, sample choice was targeted to maximize genetic diversity between individuals. A Qubit dsDNA assay was used to determine the concentration of each gDNA sample. For each cross, 3 μg of an equimolar amount of DNA of the 30 pigs (100 ng for each sample) was prepared (Pools DU and HY).

### Exome capture and massively parallel sequencing

To prepare NGS libraries, we followed the SureSelectXT Target Enrichment System for Illumina Paired-End Sequencing Library (Agilent) protocol specifications starting from 3 μg of pooled DNA. DNA was sheared by using Bioruptor (Diagenode) to obtain fragments of ~250 bp. Exome capture was performed by using a SureSelect Human All-Exon V5 kit (Agilent), which targets ~50 Mb of coding regions of human genes. Hybridization and capture were performed separately for each sample library prepared. Sequencing was performed on Illumina’s HiSeq 2000 at IGA Technology Services (Udine, Italy) using a paired-end approach with runs of 100 bp.

### Sequence alignment, variant calling, and annotation

The paired-end reads were first trimmed (minimum mean quality value to accept a trimmed sequence: 20, minimum sequence length after trimming: 50) in order to remove lower base quality data with ERNE (Extended Randomized Numerical alignEr) [9]. Alignment on the *Sus scrofa* reference genome (NCBI Sscrofa10.2) was performed with BWA (Burrows-Wheeler Aligner) [10]. Only uniquely mapping reads were selected by proprietary script. PCR and optical duplicates were removed from all alignments with Picard (http://broadinstitute.github.io/picard/). Picard Alignment and Hybrid Selection Metrics were used to generate high-level metrics about the library insert size, the alignment of reads and to obtain adequate depth of coverage specific in the pig’s exome regions within each alignment file. Coverage levels were assessed for the exonic regions and for CDS for both pools of pigs using all the available raw reads (Full dataset). For HY, the coverage levels were also calculated using a subset of 100 and 60 millions of raw reads (Restricted dataset), randomly selected using the software seqtk (https://github.com/lh3/seqtk).

Variant (SNP and indel) calling was performed with GATK (Genome Analysis Toolkit) [11]. The GATK local realignment tool was first used to locally realign reads such that the number of mismatching bases was minimized across all the reads. The GATK UnifiedGenotyper was used for variant calling. Variant Filtration was used for hard-filtering of variant calls based on the following criteria: filter out variants if located within a cluster where three or more calls are made in a 10 bp window (clusterWindowSize 10); filter out variant if there are at least four alignments with a mapping quality of zero (MQ0 > = 4) and if the proportion of alignments mapping ambiguously corresponds to 1/10th of all alignments ((MQ0/(1.0 * DP)) > 0.1, where DP is the total (unfiltered) depth over all samples); filter out variants which are covered by less than 10 reads (DP < 10); filter out variants having a low quality score (Q < 50); filter out variants with low variant confidence over unfiltered depth of non-reference samples (QD < 1.5); filter out variants based on strand bias using Fisher's exact test: FS > 60.0 for SNP calling, FS > 200.0 for indel calling. Passing-filter variants were annotated using the Annovar software tool [12]. A graphical representation of single nucleotide variants (SNVs) over the genome was carried out with Circos software [13].

### Validation and statistical analysis

The Receiver Operating Characteristic (ROC) curve was used to assess the ability to correctly identify segregated SNVs in the pooled samples for each crossbreed. For the 60 pigs, a total of 213 SNVs that resulted in segregation in the exome capture experiment were randomly selected and genotyped using the Sequenom MassARRAY^®^ system. For the Sequenom data, we assigned a value of 0 or 1 for the SNVs below or above a defined threshold of minor allele frequency (MAF). The selected thresholds of MAF were 0.01, 0.05, 0.1, 0.15 and 0.2. This method attempted to identify an optimal cutoff value in allele frequencies that was able to minimize the errors. The ROC curve plots true-positive rate (sensitivity) against its false-positive rate (1-specificity) for continuously changing cutoffs over the whole possible range of test results [14]. Statistical analysis was performed using SPSS statistical software, version 16.0 (SPSS Inc., Chicago, IL, USA). The area under the curve (AUC) of the ROC curve was determined to assess the probability that the model will assign higher prediction likelihood to SNVs that were correctly predicted than to those that were not. The accuracy (ACC) of the method was also calculated as: ACC = (∑true positives + ∑true negatives)/(∑positives + ∑negatives).

## Results and Discussion

This research was focused on finding a useful and cost-effective method for the identification of SNVs of two crossbreeds of pigs differing for productive responses. The analysis of variance performed on the whole population (DU = 1032 observations; HY = 1011 observations) showed that HY crosses had an ADG at 10 weeks significantly higher than DU pigs (P < 0.001; estimated marginal mean and standard error: 384 ± 3.8 g for HY and 359 ± 2.6 g for DU), while carcass weight was higher (P < 0.001) for DU (estimated marginal mean and standard error: 142.1 ± 0.4 kg) compared to HY (estimated marginal mean and standard error: 138.6 ± 0.6 kg). Other significant dissimilarities (P< 0.001) were observed for lean percentage (estimated marginal mean and standard error: 48.8 ± 0.1% for DU and 49.8 ± 0.1% for HY), and backfat thickness (estimated marginal mean and standard error: 29.1 ± 0.19 mm for DU and 27.0 ± 0.28 mm for HY), confirming the different productive performances and genetic makeup of two crossbreeds. Further data are reported in S1 and S2 Tables. From the statistical analysis a significant effect of farm (P< 0.001) was almost always calculated, indicating that other factors, among the other live weight at weaning, length of the growing and fattening periods, diets and plan of nutrition, farm management have played a role in determining the productive response of the pigs and the carcass quality.

We generated 140.2 M and 162.5 M of raw reads for Pool DU and Pool HY, respectively. Mean coverage of *Sus scrofa* for all exonic regions (ENSEMBL Sus_scrofa.Sscrofa10.2.73.gtf) was 89.33X and 97.56X for Pool DU and Pool HY, respectively (Table 1), while the mean coverage of coding sequence (CDS) exons was 129.97X and 141.77X for Pool DU and Pool HY, respectively.

**Table 1:** Summary for sequence volume, mapping and coverage for the exonic regions and for CDS captured with human probes (Agilent SureSelect Human All Exon V5 kit), targeting ~50 Mb of coding regions for two pools of pigs.

	Full dataset^a^				Restricted dataset^b^			
	*Exons*	*Exons*	*CDS*	*CDS*	*Exons*	*CDS*	*Exons*	*CDS*
Pool	DU	HY	DU	HY	HY	HY	HY	HY
Gbp	14.0	16.3	14.0	16.3	10.0	10.0	6.0	6.0
% Mapped	89.1	85.6	89.1	85.6	85.6	85.6	85.6	85.6
% Duplicates	12.1	17.7	12.1	17.7	9.4	9.4	7.3	7.3
% Mapped on exons	64.0	66.0	62.8	64.4	66.0	64.3	66.0	64.3
Mean Exon Coverage (x)	89.3	97.6	130.0	141.8	58.7	94.9	41.6	60.5
*Coverage % at*:								
2X	0.75	0.75	0.86	0.86	0.71	0.85	0.67	0.83
10X	0.58	0.59	0.78	0.79	0.56	0.76	0.52	0.72
20X	0.52	0.53	0.72	0.74	0.49	0.69	0.43	0.62
30X	0.48	0.49	0.68	0.69	0.44	0.63	0.37	0.53

^a^Full dataset refers to all the available raw reads;

^b^Restricted dataset refers to a subset of 100 and 60 millions of raw reads, randomly selected.

The experimental data are fully available on NCBI Sequence Read Archive (SRA) with accession number SRX1046652.

Thirty-five percent of aligned bases uniquely mapped to off-target regions. However, we believe that the efficiency of heterologous enrichment (human-on-pig) was still satisfactory, considering that analogous exome enrichment (human-on-human) yields ~20% of off target bases [15, 16].

Human Agilent exome enrichment technology is highly performant in terms of coverage efficiency as a function of sequencing depth. Fifty million reads are sufficient to cover about 95% of human target bases with at least 10X depth [16], enabling confident variant calling in these regions [17]. By applying the same human exome probe set in swine, at a sequencing depth of 160 million reads, we were able to achieve ≥ 10X coverage for 59% of all pig’s exons and 79% of CDS exons (Table 1).

**Fig 1:**
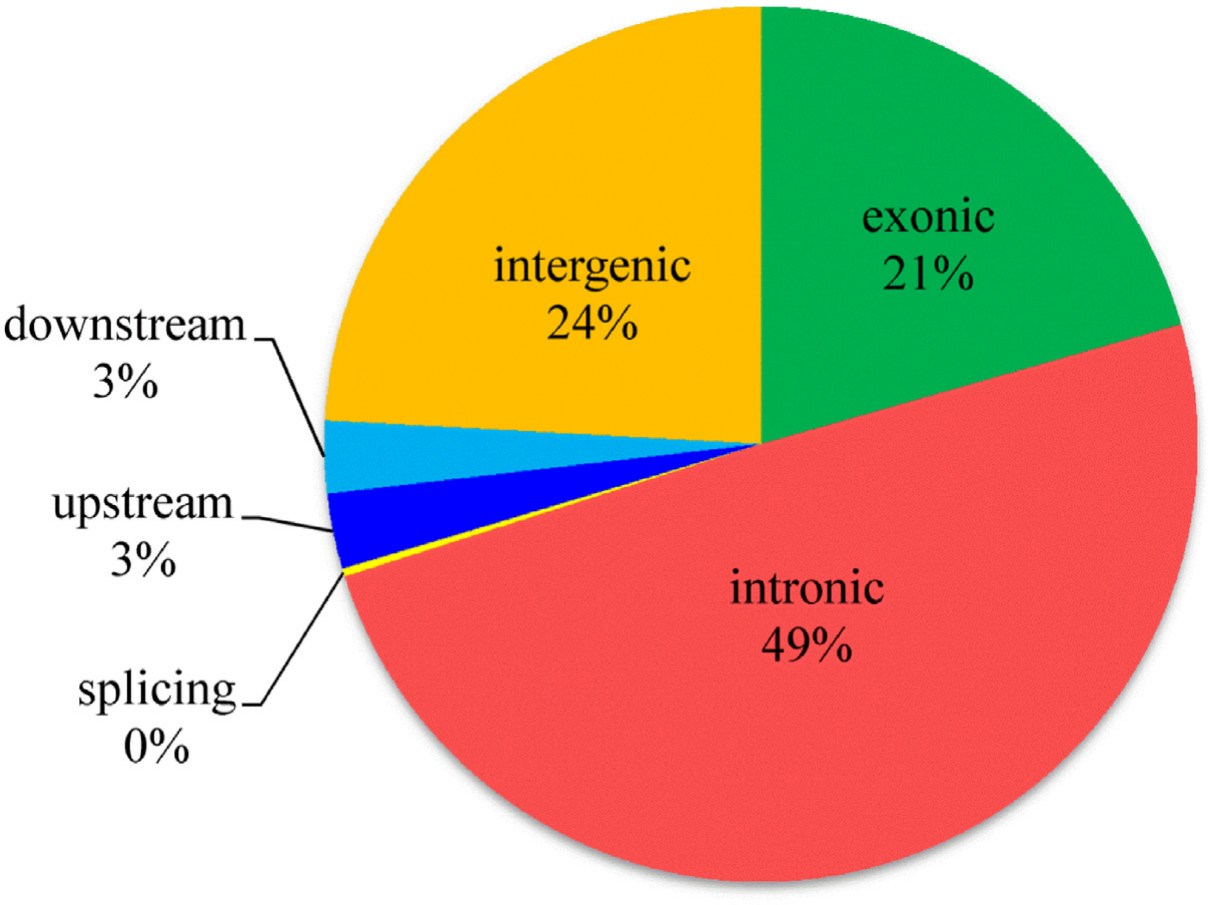
Localization of Single Nucleotide Variants found in the pig genome using a heterologous enrichment based on human probes.

In order to evaluate the variation of target coverage in relation to the number of reads, two random subsets of reads of pool HY, each constituted by 10.0 Gbp and 6.0 Gbp of the total sequenced, were used. The calculated values pinpointed that the decrease in the number of reads did not significantly reduce the portion of the covered target. Differences in coverage between chromosome regions can be related to nucleotide composition, which has been shown to bias sequencing efficiency [18]. Consequently, coverage may be artificially low for sequences with high GC or AT content [16]. The reduction of the full dataset from 16.0 Gbp to 6.0 Gbp caused a reduction of target covered ≥ 10X only of 0.07 both for exons (from 0.59 to 0.52) and CDS exons (from 0.79 to 0.72), indicating the rapid saturation of the system with very few additional regions being enriched with a substantial increase in read counts. Furthermore, this simulation indicates that the sequencing depth should be adapted according to the scope of the analysis, evaluating costs and benefits, since more sequencing would provide little additional information. Exome sequencing of individuals in pool requires a higher sequencing effort in order to discover rare or private DNA variants in a pool, while for a single individual sequencing, lower coverages can probably be used. However, it is always recommended to do the saturation analyses as an attempt to calculate the required depth at which sequencing must be carried out [19]. By comparing our sequencing data with the reference Sscrofa10.2 assembly, we identified several hundreds of thousands of putative DNA variants and, after applying the hard filtering criteria as described in the ‘Materials and methods’ section, a total of 232,530 SNVs remained (S3 Table). Among these SNVs, 23,388 SNVs segregated only in DU, while 9,784 SNVs in HY. Exome enrichment via hybridization captures exons as well as their flanking regions. In order to maximize the number of variants across DU and HY crosses, variant calling was performed across the whole pig’s genome, including also intronic and intergenic regions. Among variants, 20.6% were exonic and 49.5% intronic. About 2.8% were located in 5’ UTR, 2.7% in 3’ UTR, while 0.3% SNVs resulted as splicing variants. The remaining polymorphisms (24.1%) were mapped in intergenic regions (Fig 1). From our previous analysis (results not shown), a comparison with homologous (human-on-human) enrichment via hybridization performed using the Illumina Nextera Rapid kit evidenced that at mean coverage of 77X, 30% of discovered variants were located on exons, while 58% of SNVs were on introns. These values are comparable to present results, suggesting that the low percentage of exonic SNVs was not dependent from exon coverage levels, but was probably related to the variant calling strategy. This latter was carried out on probes extended of 200 b.p. downstream and upstream of exonic regions.

Variant functional annotation showed that 15378 of variants are nonsynonymous. This is comparable to what was observed for human whole-exome sequencing (WES) which contains about 10 000 nonsynonymous variants per individual exome, depending on ethnicity and calling methods applied [20].

**Fig 2:**
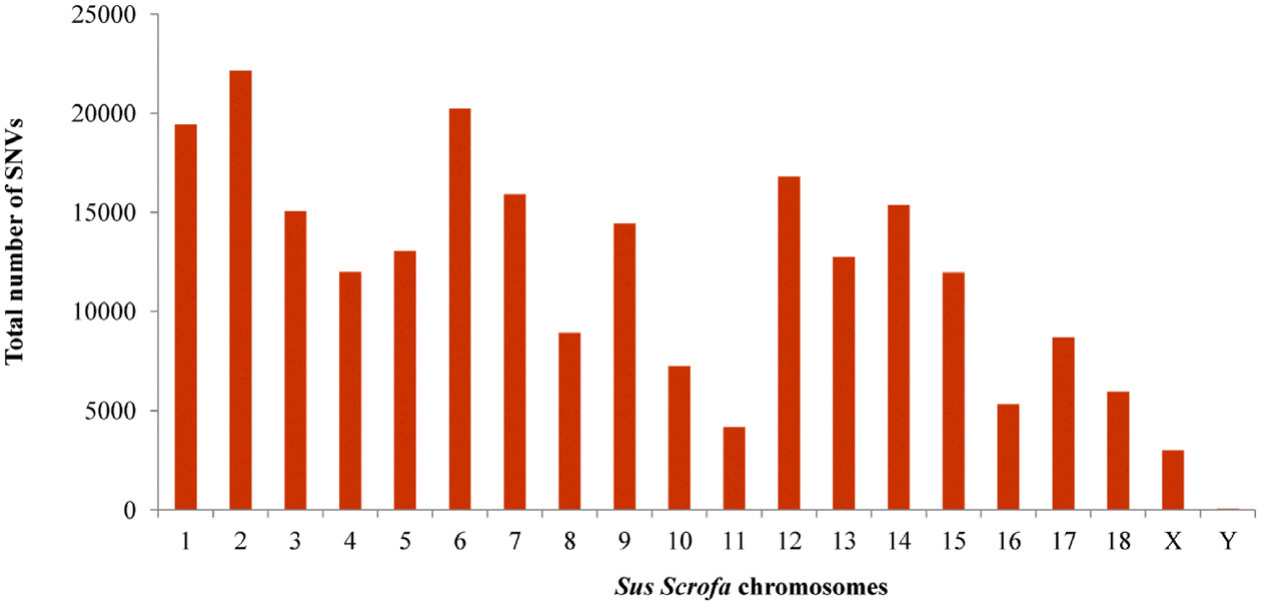
Total number of Single Nucleotide Variants for each chromosome detected in two pools of pigs using a heterologous enrichment.

**Fig 3:**
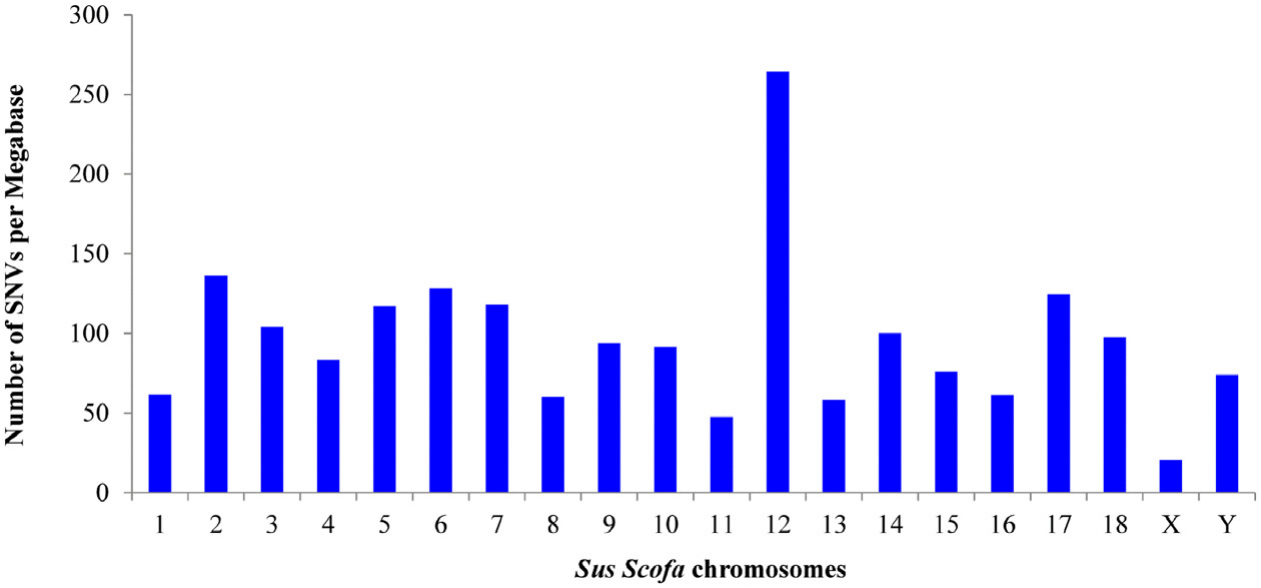
Number of Single Nucleotide Variants per Megabase for each chromosome detected in two pools of pigs using a heterologous enrichment.

**Fig 4:**
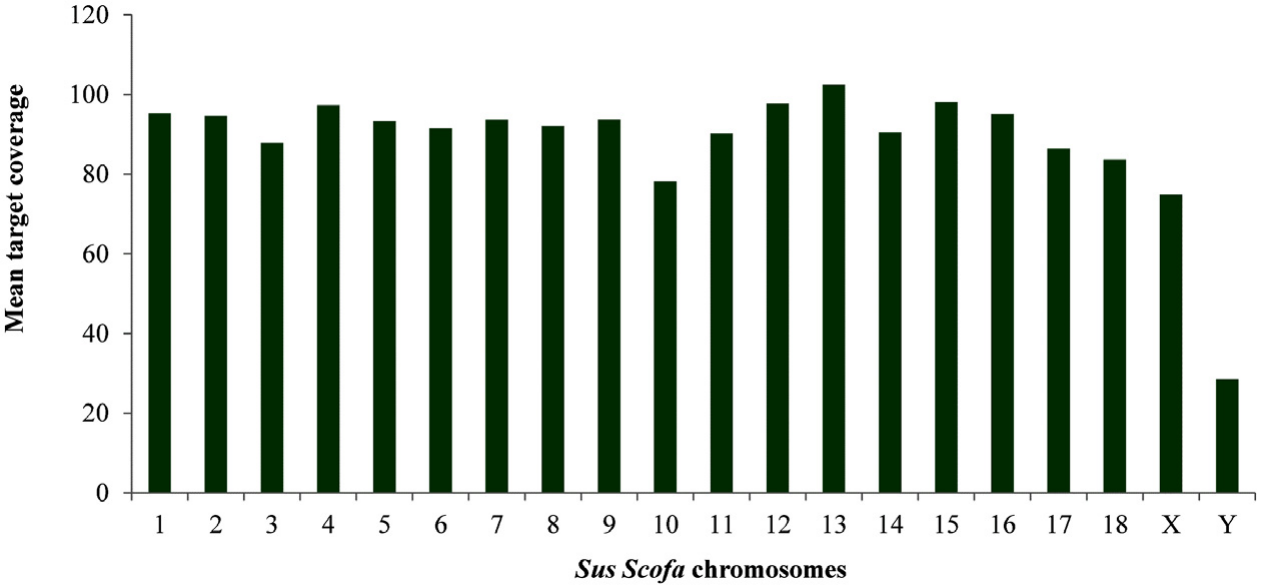
Mean target coverage of exonic regions detected in two pools of pigs using a heterologous enrichment.

The highest number of variants was found in SSCs 1, 2 and 6 (Fig 2). The number of SNVs found per chromosome depends on the level of coverage of the region, the SSC size and the genetic differences between the crossbreeds. In order to highlight regions of the genome with low and high number of DNA variants, the density of SNVs per MB of each chromosome was also calculated (Fig 3). The highest density in the autosomes was found in SSC 12, whereas the lowest was present in SSC 11; the X chromosome showed the lowest density, probably reflecting a low coverage level of this chromosome (Fig 4). However, it is likely that SNV densities of the exome across chromosomes also reflect their gene density, which has the lowest values for SSC 1, SSC 8, SSC1, SSC 13 and SSC 16 [21].

**Fig 5:**
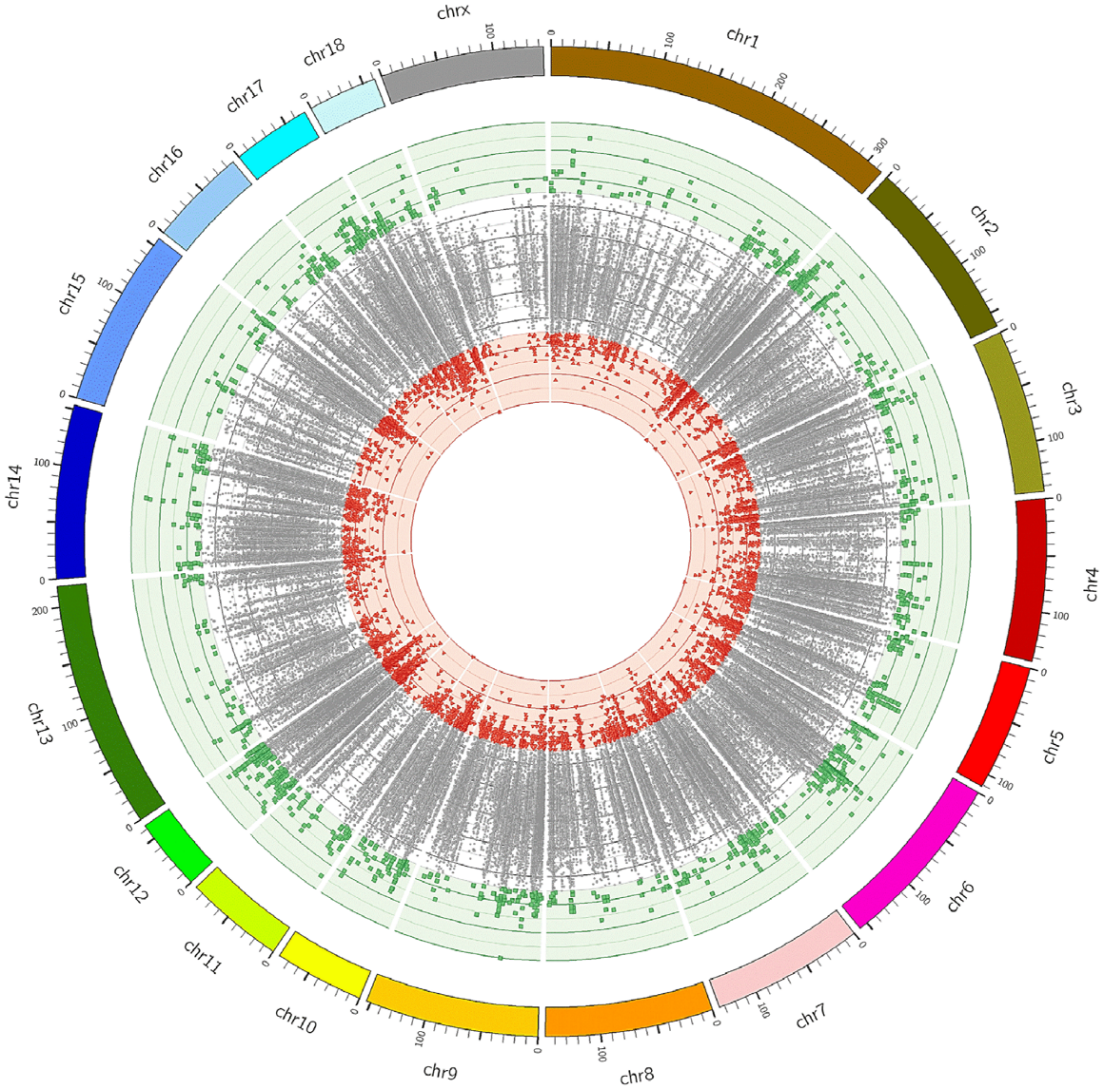
Visualization with Circos software of differences in allele frequencies between pool DU and pool HY of pigs. The spots within circle represent the SNVs mapped on each chromosome and the distance between the inner and the outer circles indicates the difference in frequency of the reference allele between DU and HY. Therefore, SNVs with a difference > +0.5 between crossbreeds are placed in the outer layer, whereas those SNVs with a difference < –0.5 are located in the inner layer. Variants with allelic difference included between ± 0.5 are reported in the intermediate layer.

For a graphical representation of found variants, a visualization of differences in allele frequencies between crossbreeds was carried out with Circos software (Fig 5). The spots within the circle represent the SNVs mapped on each chromosome and the distance between the inner and the outer circles indicates the difference in frequency of the reference allele between pools DU and HY. Therefore, SNVs with a difference > +0.5 between crossbreeds were placed in the outer layer, whereas those SNVs with a difference < –0.5 were located in the inner layer. Variants with allelic difference included between ± 0.5 were reported between the outer and inner circles.

**Fig 6:**
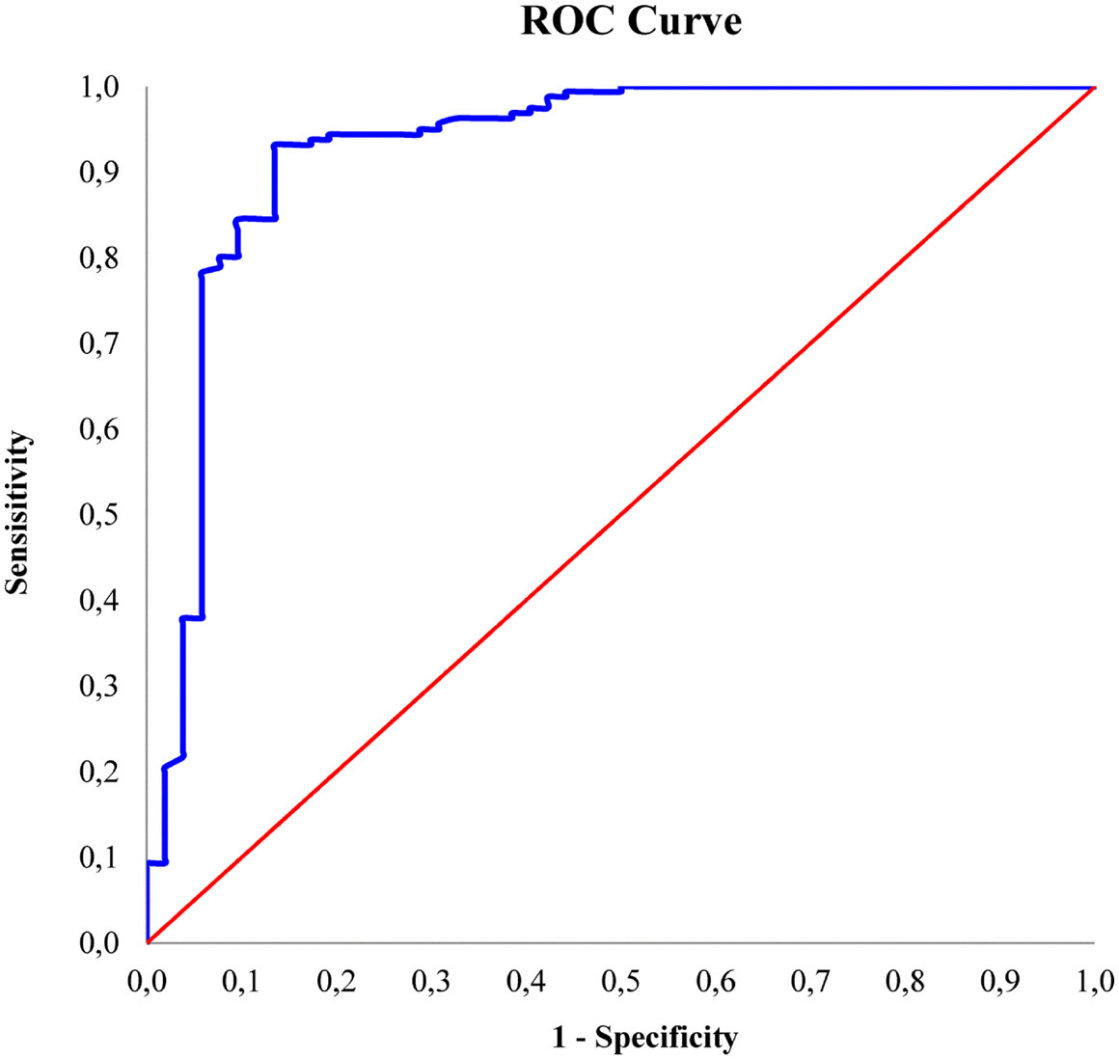
Receiver operating characteristic (ROC) curve. An example of Receiver operating characteristic (ROC) analysis for the prediction of ‘true segregating SNVs’ in the HY population using the allele frequencies in the exome capture experiment at a MAF threshold of 0.15.

The allele frequencies obtained for the 213 SNVs with Sequenom were used to validate the frequencies calculated from the exome sequencing using the ROC curve technique. The curve plots the true positive rate (sensitivity) on the Y axis against the false positive rate (1—specificity) on the X axis at different thresholds. The ROC technique is already used in biomedical science to identify threshold to cluster population in positive (affected) and negative (unaffected) individuals and the test is considered reliable when the AUC is higher than 0.80 [22]. The optimal prediction is obtained when the ROC curve drifts to the upper left corner (coordinate x = 0; y = 1), corresponding to 100% sensitivity (no false negatives) and 100% specificity (no false positives). We set MAF thresholds of 0.01, 0.05, 0.10, 0.15 and 0.20 for the test variable, attributing a value of 0 or 1 for MAF values lower and higher than the different thresholds. The AUC values from the ROC curve for the selected thresholds (Table 2) were optimal at a MAF level < 0.10 for DU pig populations and < 0.15 for the HY pool The ROC curve for HY at the optimal threshold is reported in Fig 6. At the selected thresholds, the prediction ACC of the model was also high: 0.953 for DU and 0.906 for HY, although we found high AUC and ACC values for all the selected thresholds. As can be seen in Table 2, AUC and ACC were not linearly related with MAF thresholds and this can be due to the number of SNVs close to the selected thresholds, thus influencing the number of false positives and false negatives.

**Table 2:** Receiver operating characteristic (ROC) analysis. Prediction of ‘true segregating SNVs’ at increasing MAF thresholds.

Pool	MAF	Positive	Negative	AUC^a^	s.e.^b^	P^c^	ACI 95%^d^		ACC^g^
							LB^e^	UP^f^	
**DU**	0.01	196	17	0.833	0.058	5.4E-6	0.719	0.947	0.925
	0.05	182	31	0.928	0.034	2.7E-14	0.862	0.994	0.934
	0.10	175	38	0.938	0.028	2.5E-17	0.884	0.993	0.953
	0.15	161	52	0.927	0.026	2.3E-20	0.875	0.978	0.892
	0.20	150	63	0.913	0.029	2.1E-21	0.855	0.970	0.915
**HY**	0.01	188	25	0.800	0.057	1.1E-6	0.688	0.912	0.939
	0.05	178	35	0.811	0.051	6.1E-9	0.712	0.911	0.915
	0.10	160	53	0.882	0.034	7.6E-17	0.815	0.948	0.892
	0.15	142	71	0.894	0.03	7.2E-21	0.834	0.954	0.906
	0.20	130	83	0.891	0.028	7.4E-22	0.835	0.946	0.869

^a^AUC = Area Under Curve;

^b^s.e. = standard error;

^c^P = asymptotic P-value under the null hypothesis that AUC = 0.5;

^d^ACI 95% = Asymptotic 95% Confidence Interval;

^e^LB = Lower Bound;

^f^UP = Upper Bound;

^g^ACC = Accuracy

To further compare the results between Sequenom and exome capture, a Spearman correlation test was also applied, obtaining values of 0.73 and 0.74 for DU and HY, respectively.

## Conclusions

The obtained results indicate that use of a human design exon-capture system can provide a satisfactory enrichment of pig gene regions for the identification of novel DNA variants in protein-coding genes. In particular, after applying suitable filtering criteria, the researchers are able to find variants with the most divergent allele frequencies at the pool levels that can be later used for biodiversity studies and for the selection trials of phenotypic traits of relevance. Our data indicate that the analysis of pooled samples can be considered an alternative strategy to the resequencing of a large number of individuals if the SNV discovery is the main aim of the research. With this approach, scientists who are planning exome sequencing experiments can avoid to perform expensive and time-consuming analysis.

A direct comparison between homologous and heterologous enrichment in pig is not possible because the available data were produced with different techniques and populations. However it is likely that the human-on-pig approach can be applied for the successful identification of SNVs, while a species-specific enrichment is more appropriate if structural variations, such as large indels, inversions and copy number variations (CNV) as well as small RNAs are the focus of the study.

## Supporting Information

S1 Table Estimated marginal means of growth performances of pig crossbreeds used in the study. (DOCX)

S2 Table Estimated marginal means of carcass characteristics of pig crossbreeds used in the study. (DOCX)

S3 Table Single nucleotide variants (SNVs) discovered in comparison to the reference genome. (ZIP)

## References

[1] European Commission (2014) Publication, pursuant to Article 18(2) of Commission Regulation (EC) No 1898/2006, of the single document on a designation of origin or a geographical indication registered under Commission Regulation (EC) No 1107/96 in accordance with Article 17 of Council Regulation (EEC) No 2081/92. Official Journal of the European Union C 188/24.

[2] Guiatti D, Sgorlon S, Stefanon B. (2013) Association analysis between single nucleotide polymorphisms in the promoter region of LEP, MYF6, MYOD1, OPN, SCD genes and carcass traits in heavy pigs. Italian Journal Animal Science, 12:77–82.

[3] Herrero-Medrano JM, Megens HJ, Groenen MA, Bosse M, Pérez-Enciso M, Crooijmans RP. (2014) Whole-genome sequence analysis reveals differences in population management and selection of European low-input pig breeds. BMC Genomics. 15:601 10.1186/1471-2164-15-601 25030608 PMC4117957

[4] Ramos AM, Crooijmans RP, Affara NA, Amaral AJ, Archibald AL, Beever JE, et al (2009) Design of a high density SNP genotyping assay in the pig using SNPs identified and characterized by next generation sequencing technology. PLoS One, 4:e6524 10.1371/journal.pone.0006524 19654876 PMC2716536

[5] Ng SB, Buckingham KJ, Lee C, Bigham AW, Tabor HK, Dent KM, et al (2010) Exome sequencing identifies the cause of a mendelian disorder. Nat Genet, 42(1):30–35. 10.1038/ng.499 19915526 PMC2847889

[6] Robert C, Fuentes-Utrilla P, Troup K, Loecherbach J, Turner F, Talbot R, et al (2014) Design and development of exome capture sequencing for the domestic pig (Sus scrofa). BMC Genomics. 15:550 10.1186/1471-2164-15-550 24988888 PMC4099480

[7] Jin X, He M, Ferguson B, Meng Y, Ouyang L, Ren J, et al (2012) An Effort to Use Human-Based Exome Capture Methods to Analyze Chimpanzee and Macaque Exomes. PLoS ONE 7(7). 10.1371/journal.pone.0040637 PMC3407233 22848389

[8] Khlopova NS, Stefanon B, Guiatti D, Glazko TT, Glazko VI. (2012) Single Nucleotide Polymorphism of promoters of candidate genes controlling porcine productivity indices. Russian Agricultural Sciences, 38: 311–317.

[9] Del Fabbro C, Scalabrin S, Morgante M, Giorgi FM. (2013) An Extensive Evaluation of Read Trimming Effects on Illumina NGS Data Analysis. PLoS One 8(12):e85024 10.1371/journal.pone.0085024 24376861 PMC3871669

[10] Li H, Durbin R. (2009) Fast and accurate short read alignment with Burrows-Wheeler Transform. Bioinformatics, 25:1754–60. 10.1093/bioinformatics/btp324 19451168 PMC2705234

[11] McKenna A, Hanna M, Banks E, Sivachenko A, Cibulskis K, Kernytsky A, et al (2010) The Genome Analysis Toolkit: A MapReduce framework for analyzing next-generation DNA sequencing data. Genome Research, 20(9), 1297–1303. 10.1101/gr.107524.110 20644199 PMC2928508

[12] Wang K, Li M, Hakonarson H. (2010) ANNOVAR: Functional annotation of genetic variants from next-generation sequencing data Nucleic Acids Research, 38:e164 10.1093/nar/gkq603 20601685 PMC2938201

[13] Krzywinski M, Schein JE, Birol I, Connors J, Gascoyne R, Horsman D, et al (2009) Circos: an Information Aesthetic for Comparative Genomics. Genome Res 19:1639–1645. 10.1101/gr.092759.109 19541911 PMC2752132

[14] Tsairidou S, Woolliams JA, Allen AR, Skuce RA, McBride AH, Wright DM, et al (2014) Genomic Prediction for Tuberculosis Resistance in Dairy Cattle. PLoS One 9(5):e96728 10.1371/journal.pone.0096728 24809715 PMC4014548

[15] Clark MJ, Chen R, Lam HY, Karczewski KJ, Chen R, Euskirchen G, et al (2011) Performance comparison of exome DNA sequencing technologies. Nat Biotechnol. 29(10):908–14. 10.1038/nbt.1975 21947028 PMC4127531

[16] Chilamakuri CS, Lorenz S, Madoui MA, Vodák D, Sun J, Hovig E, et al (2014) Performance comparison of four exome capture systems for deep sequencing. BMC Genomics 15:449 10.1186/1471-2164-15-449 24912484 PMC4092227

[17] Fuentes Fajardo KV, Adams D, Mason CE, Sincan M, Tifft C, Toro C, et al (2012) Detecting false-positive signals in exome sequencing. Human mutation 33: 609–613. 10.1002/humu.22033 22294350 PMC3302978

[18] Gnirke A, Melnikov A, Maguire J, Rogov P, LeProust EM, Brockman W, et al (2009) Solution hybrid selection with ultra-long oligonucleotides for massively parallel targeted sequencing. Nat Biotechnol. 27:182–189. 10.1038/nbt.1523 19182786 PMC2663421

[19] Sims D, Sudbery I, Ilott NE, Heger A, Ponting CP (2014) Sequencing depth and coverage: key considerations in genomic analyses. Nat Rev Genet. 15(2):121–32 10.1038/nrg3642 24434847

[20] Rabbani B, Tekin M, Mahdieh N (2014) The promise of whole-exome sequencing in medical genetics. J Hum Genet. 59(1):5–15. 10.1038/jhg.2013.114 24196381

[21] Steibel JP, Bates RO, Rosa GJM, Tempelman RJ, Rilington VD, Ragavendran A, et al (2011) Genome-Wide Linkage Analysis of Global Gene Expression in Loin Muscle Tissue Identifies Candidate Genes in Pigs. PLoS ONE 6(2): e16766 10.1371/journal.pone.0016766 21346809 PMC3035619

[22] Tekola Ayele F, Hailu E, Finan C, Aseffa A, Davey G, Newport MJ, et al (2012) Prediction of HLA class II alleles using SNPs in an African population. PLoS One. 7(6):e40206 10.1371/journal.pone.0040206 22761960 PMC3386230

