# Supplementary material for: Exome Capture with Heterologous Enrichment in Pig (Sus scrofa)
Source: PLoS One. 2015 Oct 2;10(10):e0139328. doi: 10.1371/journal.pone.0139328 (PMC4592256; doi:10.1371/journal.pone.0139328)
Supplement: S1 Table — (DOCX) [file pone.0139328.s001.docx]

**S1 Table. Estimated marginal means of growth performances of pig crossbreeds used in the study.**

The pigs used in this research belong to Italian Duroc X Large White crossbreeds (DU) and Commercial hybrid X Large White crossbreeds (HY). The Duroc boars and the Large White sows belonged to genetic pure lines selected by National Association of Pig Breeders (ANAS, Rome, Italy). These crosses are widely used in Italian heavy pig production chain and in the table the productive performances collected on 3 farms from 2043 animals are reported. The statistical model used was ANOVA with Farm and Crossbreed as fixed effects.

|  |  |  |  |  |  |  |  |  |  |
| --- | --- | --- | --- | --- | --- | --- | --- | --- | --- |
| **Farm** | **Crossbreed** | **W1^a^** | **A1^b^** | **W2^c^** | **A2^d^** | **W3^e^** | **AS^f^** | **ADG1^g^** | **ADG2^h^** |
|  |  |  |  |  |  |  |  |  |  |
| F1 | DU | 7.1 | 29 | 20.7 | 71 | 169.4 | 297 | 322 | 652 |
|  | HY | 7.2 | 28 | 20.8 | 70 | 166.1 | 301 | 321 | 631 |
|  |  |  |  |  |  |  |  |  |  |
| F2 | DU | 7.0 | 27 | 24.2 | 73 | 169.6 | 295 | 358 | 638 |
|  | HY | 7.1 | 31 | 29.8 | 82 | 161.0 | 279 | 457 | 663 |
|  |  |  |  |  |  |  |  |  |  |
| F3 | DU | 8.0 | 29 | 23.6 | 70 | 170.6 | 268 | 398 | 736 |
|  | HY | 8.2 | 27 | 23.9 | 70 | 166.8 | 267 | 376 | 719 |
|  |  |  |  |  |  |  |  |  |  |
|  |  |  |  |  |  |  |  |  |  |
| Average | DU | 7.2 | 27 | 22.8 | 72 | 169.9 | 286 | 359 | 675 |
|  | HY | 7.6 | 29 | 25.8 | 75 | 164.7 | 282 | 384 | 671 |
|  |  |  |  |  |  |  |  |  |  |
| Average | DU and HY | 7.3 | 28 | 24.3 | 74 | 167.3 | 284 | 372 | 673 |
| MSE^i^ |  | 2.2 | 21 | 19.6 | 47 | 225.8 | 292 | 5339 | 5286 |
|  |  |  |  |  |  |  |  |  |  |
| Effects |  |  |  |  |  |  |  |  |  |
| *Farm* |  | *** | ** | *** | *** | *** | *** | *** | *** |
| *Crossbreed* |  | *** | *** | * | ns | *** | *** | *** | ns |
| *Farm x Crossbreed* |  | * | ns | ns | ns | ** | * | ** | * |
|  |  |  |  |  |  |  |  |  |  |

**Legend** ^a^W1 = mean weight (kg) at weaning; ^b^A1 average days at weaning; ^c^W2 = mean weight (kg) at 10 weeks; ^d^A2 average days at 10 weeks; ^e^W3 = mean weight (kg) at slaughter; ^f^AS average days at slaughter; ^g^ADG1 = average daily gain (g) from weaning (W1) to 10 weeks (W2); ^h^ADG2 = average daily gain (g) from 10 weeks (W2) to slaughter (W3); ^i^MSE = Mean Square Error

**Analysis of variance**

Statistical significance of effects: *** = p<0.001; ** = p<0.01; * = p<0.05; ns = p ≥0.05
