# Supplementary material for: Exome Capture with Heterologous Enrichment in Pig (Sus scrofa)
Source: PLoS One. 2015 Oct 2;10(10):e0139328. doi: 10.1371/journal.pone.0139328 (PMC4592256; doi:10.1371/journal.pone.0139328)
Supplement: S2 Table — (DOCX) [file pone.0139328.s002.docx]

**S2 Table. Estimated marginal means of carcass characteristics of pig crossbreeds used in the study.**

The animals used in this research belong to Italian Duroc X Large White crossbreeds (DU) and Commercial hybrid X Large White crossbreeds (HY). The Duroc boars and the Large White sows belonged to genetic pure lines selected by National Association of Pig Breeders (ANAS, Rome, Italy). These crosses are widely used in Italian heavy pig production chain. In the table, the average carcass parameters collected on 3 farms from 2043 animals are reported. The statistical model used was ANOVA with Farm and Crossbreed as fixed effects.

|  |  |  |  |  |  |  |  |
| --- | --- | --- | --- | --- | --- | --- | --- |
| **Farm** | **Crossbreed** | **CW^a^** | **FOM^b^** | **DMT^c^** | **BFT^d^** | **TW^e^** | **TTW^f^** |
|  |  |  |  |  |  |  |  |
|  |  |  |  |  |  |  |  |
| F1 | DU | 139.4 | 48.8 | 65.0 | 28.8 | 16.7 | 14.4 |
|  | HY | 137.6 | 49.6 | 67.1 | 27.6 | 16.3 | 14.0 |
|  |  |  |  |  |  |  |  |
| F2 | DU | 143.7 | 49.7 | 67.8 | 27.7 | 17.8 | 15.6 |
|  | HY | 136.9 | 51.4 | 68.5 | 24.0 | 16.2 | 14.0 |
|  |  |  |  |  |  |  |  |
| F3 | DU | 143.4 | 47.9 | 65.4 | 30.7 | 17.2 | 15.1 |
|  | HY | 141.5 | 48.3 | 64.5 | 29.4 | 16.3 | 14.4 |
|  |  |  |  |  |  |  |  |
| Average | DU | 142.1 | 48.8 | 66.1 | 29.1 | 17.2 | 15.1 |
|  | HY | 138.6 | 49.8 | 66.7 | 27.0 | 16.3 | 14.1 |
|  |  |  |  |  |  |  |  |
| Average | DU and HY | 140.4 | 49.3 | 66.4 | 28.0 | 16.7 | 14.6 |
| MSE^g^ |  | 157.3 | 7.6 | 49.9 | 31.9 | 2.1 | 1.3 |
|  |  |  |  |  |  |  |  |
| Effects |  |  |  |  |  |  |  |
| *Farm* |  | *** | *** | *** | *** | *** | *** |
| *Crossbreed* |  | *** | *** | ns | *** | *** | *** |
| *Farm x Crossbreed* |  | ** | ** | ns | *** | *** | ** |

**Legend**

^a^CW = carcass weight. kg; ^b^FOM = Lean cuts. % (Fat O Meter); ^c^DMT = dorsal muscle thickness. mm; ^d^BFT = Back fat thickness. mm; ^e^TW = Thigh weight. kg; ^f^TTW = Trimmed thigh weight. kg;^g^MSE = Mean Square Error

**Analysis of variance**

Statistical significance of effects: *** = p<0.001; ** = p<0.01; ns = p ≥0.05
